# An Alternative Splicing Variant of the Mixed-Lineage Leukemia 5 Protein Is a Cellular Adhesion Receptor for ScaA of Orientia tsutsugamushi

**DOI:** 10.1128/mbio.01543-22

**Published:** 2022-12-21

**Authors:** Yen Thi Hai Nguyen, Chaewon Kim, Hong-Il Kim, Yuri Kim, Sang-Eun Lee, Sunghoe Chang, Na-Young Ha, Nam-Hyuk Cho

**Affiliations:** a Department of Microbiology and Immunology, Seoul National University College of Medicine, Seoul, Republic of Korea; b Department of Biomedical Sciences, Seoul National University College of Medicine, Seoul, Republic of Korea; c Institute of Endemic Disease, Seoul National University Medical Research Center, Seoul, Republic of Korea; d Department of Physiology, Seoul National University College of Medicine, Seoul, Republic of Korea; e Biomedical Research Institute, Chungnam National University Hospital, Daejeon, Republic of Korea; f Seoul National University Bundang Hospital, Seongnam, Republic of Korea; g Wide River Institute of Immunology, Seoul National University, Hongcheon, Republic of Korea; Ohio State University

**Keywords:** scrub typhus, *Orientia tsutsugamushi*, mixed-lineage leukemia 5, adhesion, receptor, vaccine

## Abstract

Scrub typhus is a mite-borne disease caused by the obligately intracellular bacterium Orientia tsutsugamushi. We previously demonstrated that ScaA, an autotransporter membrane protein of O. tsutsugamushi, is commonly shared in various genotypes and involved in adherence to host cells. Here, we identified a mixed-lineage leukemia 5 (MLL5) mammalian trithorax group protein as a host receptor that interacts with ScaA. MLL5, identified by yeast two-hybrid screening, is an alternative splicing variant of MLL5 (vMLL5) which contains 13 exons with additional intron sequences encoding a tentative transmembrane domain. Indeed, vMLL5 is expressed on the plasma membrane as well as in intracellular compartments in eukaryotic cells and colocalized with adherent O. tsutsugamushi. In addition, ScaA-expressing Escherichia coli showed significantly increased adherence to vMLL5-overexpressing cells compared with vector control cells. We mapped the C-terminal region of the passenger domain of ScaA as a ligand for vMLL5 and determined that the Su(var)3-9, Enhancer of zeste, Trithorax (SET) domain of MLL5 is an essential and sufficient motif for ScaA binding. We observed significant and specific inhibition of bacterial adhesion to host cells in competitive inhibition assays using the C-terminal fragment of ScaA or the SET domain of vMLL5. Moreover, immunization with the C-terminal fragment of ScaA provided neutralizing activity and protective immunity against lethal challenge with O. tsutsugamushi as efficiently as vaccination with the whole passenger domain of ScaA. These results indicate that vMLL5 is a novel cellular receptor for ScaA-mediated adhesion of O. tsutsugamushi and facilitates bacterial adhesion to host cells, thereby enhancing bacterial infection.

## INTRODUCTION

Scrub typhus is a mite-borne febrile illness caused by the obligately intracellular bacterium, Orientia tsutsugamushi (1). It is transmitted to the mammalian host by the bite of trombiculid mites or chiggers at the larval stage (2). Approximately 5 to 14 days after being bitten by the infected vector, patients often develop flu-like symptoms, rash, nausea, lymphadenopathy, and an eschar at the bite site. Due to the nonspecific nature of the symptoms of the disease, treatment with appropriate antibiotics is often delayed. Hence, untreated patients can suffer from more severe symptoms, including pneumonitis, meningitis, myocarditis, acute renal failure, and acute respiratory distress syndrome. The median mortality of untreated scrub typhus is 6.0%, with a wide range of 0 to 70% (3). The traditional geographic distribution of scrub typhus, called the “tsutsugamushi triangle,” includes the Asia-Pacific rim from the southeastern part of Russia to northern Australia and mid-East Asia. Within this area of endemicity, increasing disease incidence and scrub typhus outbreaks have often been reported (1, 4). Moreover, recent reports have shown that confirmed or suspected cases of disease occur outside traditional regions of endemicity (5). Molecular genomic DNA detection and/or clinical isolation of *Orientia* pathogens has been reported in the Middle East and South Africa (6–9). Mites and small mammals infected with species closely related to O. tsutsugamushi have been detected in Africa and southern Europe. A novel *Orientia* species (Orientia chuto) was also isolated from a human patient in Dubai (6). These reports suggest that the disease is not only confined to the previously known regions of endemicity but also present on other continents (10, 11).

To prevent scrub typhus, various vaccines have been tested over the past several decades; however, there is still no commercial vaccine available. Due to antigenic variation, most vaccines evaluated in trials induced short-term immunity or provided protective immunity only for the homologous strain (12, 13). Thus, understanding the complex interactions between the host and the intracellular pathogen is crucial for identifying potential molecular targets for vaccine candidates.

O. tsutsugamushi is an obligately intracellular bacterium that must be internalized in host cells for survival. Although the precise mechanism of intracellular invasion of O. tsutsugamushi remains poorly characterized, several bacterial factors have been identified as crucial ligands for the attachment and invasion of host cells. The 56-kDa type-specific antigen (TSA56) accounts for the largest proportion of bacterial outer membrane proteins and is responsible for the interaction with host fibronectin for cellular invasion (14). After bacterial attachment, it can activate integrin-mediated signal transduction pathways that induce local actin rearrangement at the infection site (15). In addition, O. tsutsugamushi encodes six autotransporter proteins (ScaA to ScaF) in various genotypes (16, 17).

Autotransporters, the largest family of outer membrane proteins in Gram-negative bacteria, play important roles in virulence, including adhesion, invasion, biofilm formation, and cellular toxicity (18). In a previous genetic analysis, *scaA*, *scaC*, *scaD*, and *scaE* were detected in most O. tsutsugamushi DNA samples collected from 12 countries, but *scaB* and *scaF* were positive in only 33.7% and 45.5% of samples, respectively, suggesting heterogeneous conservation of the Sca family proteins in O. tsutsugamushi (17). We demonstrated that conserved ScaC potentially mediates bacterial adhesion to host cells via fibronectin-binding, whereas ScaB plays a role in invasion using unknown host receptors (19, 20). Preincubation of mammalian cell cultures with either ScaB or ScaC polypeptides has been shown to inhibit bacterial adhesion but failed to induce protective immunity in a mouse challenge model using ScaC as a vaccine antigen (21). In contrast, ScaA mediates bacterial adhesion to host cells, and antibodies against ScaA can neutralize bacterial infection of host cells. Moreover, immunization with ScaA proteins provided protective immunity in mice challenged with the homologous genotype and significantly enhanced protection against infection with heterologous genotypes (21). Given that ScaA is the most universally distributed autotransporter protein among the six Sca family members of O. tsutsugamushi and is highly potent in inducing protective immunity, further investigation of the molecular interaction of ScaA with host factors may not only provide the precise mechanism of its pathogenic role during bacterial infection but also facilitate efficient vaccine development by supporting molecular antigen design to enhance protective immunity.

In this study, we identified a variant of mixed-lineage leukemia 5 (vMLL5) mammalian trithorax group protein as a host receptor for ScaA. Overexpression of vMLL5 resulted in increased attachment of ScaA-expressing Escherichia coli to host cells. In addition, we determined the specific site of interaction between vMLL5 and ScaA using a glutathione *S*-transferase (GST) pulldown assay. The C-terminal region of ScaA was found to strongly bind to MLL5 via the SET domain and induce protective immunity against lethal challenge of O. tsutsugamushi.

## RESULTS

### Characterization of MLL5 identified from Y2H screening.

**FIG 1:**
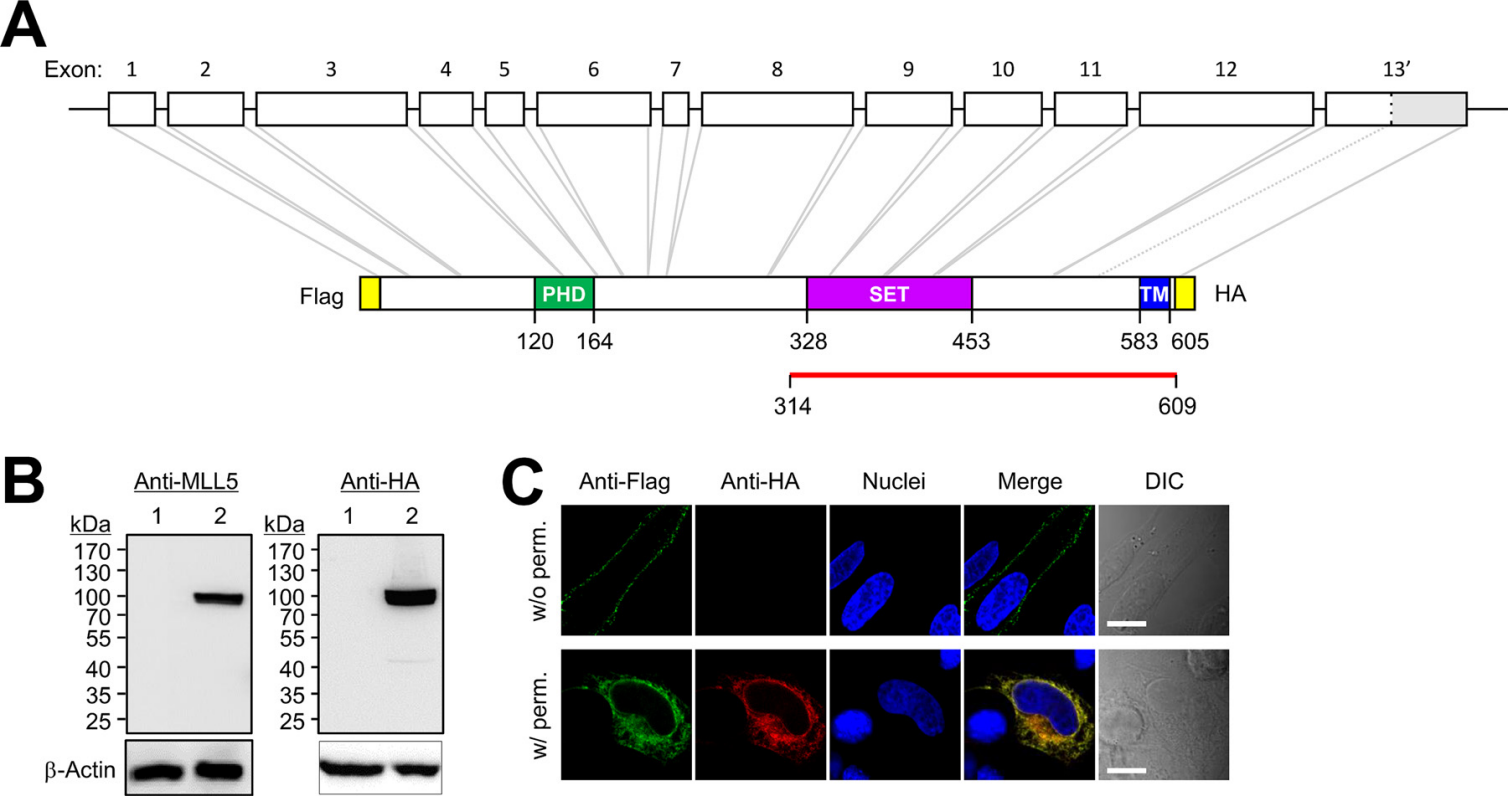
Characterization of vMLL5. (A) Schematic presentation of the vMLL5 interaction with ScaA. The domain structures were predicted using SMART software. The corresponding amino acid position of vMLL5 identified by yeast two-hybrid screening is indicated with a red line. PHD, plant homeodomain zinc finger; SET, Su(var)3-9, enhancer of zeste, and trithorax; TM, transmembrane. (B) Expression of vMLL5 was confirmed by Western blot analysis using anti-MLL5 and anti-HA antibodies. Lane 1, untransfected; lane 2, transfected. (C) Representative immunofluorescence images of cells overexpressing vMLL5. HeLa cells were transfected with vMLL5 and stained with anti-HA and anti-Flag antibodies before (w/o perm) or after (w/perm) permeabilization. Bars, 10 μm.

To identify the ScaA receptor on host cells, we performed a Y2H screening analysis using a GAL4 transcription activation domain-fused human aorta cDNA library. Using the GAL4 DNA-binding domain fused to the ScaA passenger domain (amino acids 30 to 1000 in the Boryong genotype) as a bait, we identified 12 independent positive cDNA clones (see Fig. S1 in the supplemental material). Among the putative ScaA-binding clones, lysine-specific methyltransferase 2, also known as the MLL5 protein, attracted our attention. MLL5 proteins are evolutionarily conserved trithorax groups that play roles in the regulation of homeotic gene expression, cell cycle progression, and maintenance of genomic stability (22). Although MLL5 and its variants are primarily found in nuclear or subcellular locations, NKp44L, another recently discovered MLL5 variant, is expressed on the cell surface and functions as a cellular ligand for NKp44 in natural killer cells (23). Considering that MLL5 variants have different functions depending on their location, we further characterized the sequence and cellular location of MLL5 identified in Y2H screening. The ScaA-interacting cDNA clone of MLL5 encodes the C-terminal region of a previously reported MLL5 variant sequence (Fig. 1A) (GenBank accession no. BC062583.1) (24). Using bioinformatics analysis, we found that ScaA-binding MLL5 is an alternative splicing variant of MLL5, and its gene consists of exon 1 through part of exon 13 and additional intron sequences, with a predicted protein of 609 amino acids that contains the plant homeodomain zinc finger (PHD), Su(var)3-9, the enhancer of zeste, and trithorax (SET) domain; it also has additional intron sequences predicted to encode the transmembrane domain (Fig. 1A). We designated this MLL5 isoform vMLL5.

10.1128/mbio.01543-22.1 FIG S1 ScaA-interacting host factors identified by yeast two-hybrid screening. Download FIG S1, PDF file, 0.3 MB. Copyright © 2022 Nguyen et al. 2022 Nguyen et al. https://creativecommons.org/licenses/by/4.0/ This content is distributed under the terms of the Creative Commons Attribution 4.0 International license.

To examine the cellular location of vMLL5, we generated a vMLL5 plasmid double tagged with N-terminal Flag and C-terminal hemagglutinin (HA). HeLa cells were transfected with the plasmid encoding vMLL5 and analyzed by Western blotting and confocal microscopy. As shown in Fig. 1B, immunoblot analysis showed that although its predicted molecular weight is 69 kDa, anti-MLL5 and anti-HA antibodies recognized the approximately 100-kDa vMLL5 protein. Delayed migration of another MLL5 isoform in gel electrophoresis was also observed in a previous study (25). The immunofluorescence assay detected only N-terminal Flag tag on the cellular surface but failed to detect the C-terminal HA tag in nonpermeabilized cells, indicating that vMLL5 is expressed on the plasma membrane (Fig. 1C). Membrane permeabilization of transfected cells with 0.2% Triton X-100 solution enabled us to detect both Flag and HA epitopes of vMLL5 in intracellular compartments. These results suggested that ScaA-interacting vMLL5 is expressed both on the cell surface and in subcellular regions.

### Interaction of vMLL5 with ScaA.

**FIG 2:**
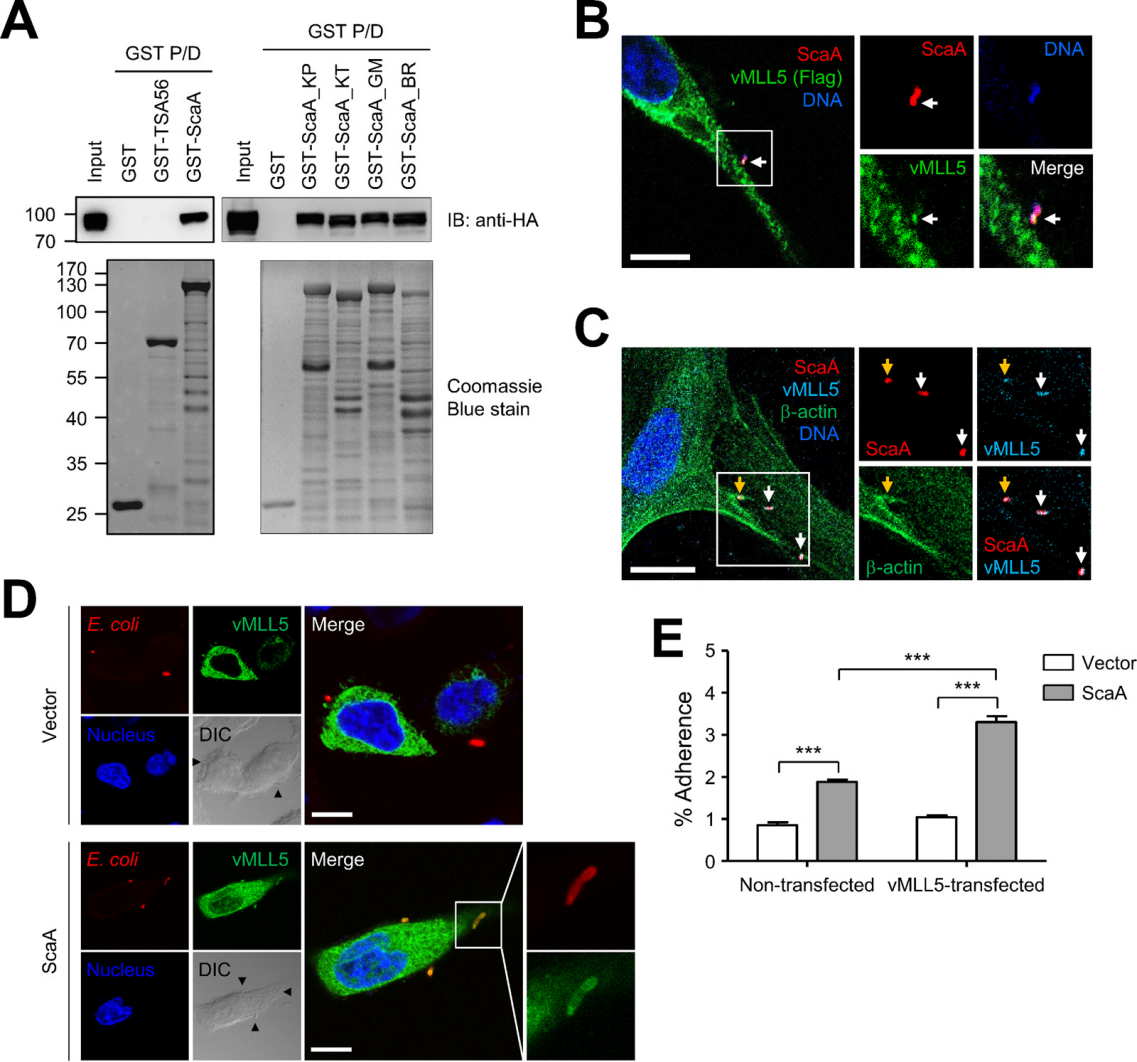
Interaction of vMLL5 and ScaA. (A) The physical interaction between ScaA and vMLL5 was examined by *in vitro* GST pulldown analysis. Purified GST fusion proteins were incubated with HEK293T cell lysate expressing full-length vMLL5. The interaction with vMLL5 protein was detected by anti-HA antibody. KP, Karp; KT, Kato; GM, Gilliam; BR, Boryong. (B) Immunofluorescence confocal microscopy of infected HeLa cells overexpressing vMLL5 showed that O. tsutsugamushi (red for ScaA and blue for bacterial DNA) colocalized with vMLL5 (green, Flag tagged) at the cell surface. Bars, 5 μm. (C) Immunofluorescence confocal microcopy of infected HUVECs showed that O. tsutsugamushi (red) colocalized with endogenous MLL5 (cyan). Actin mobilization surrounding invading O. tsutsugamushi (orange arrow) was visualized by phalloidin staining (green). White arrows indicate adherent O. tsutsugamushi without inducing actin reorganization. Bars, 10 μm. (D) HeLa cells transfected with vMLL5 were incubated with E. coli harboring empty vector or pScaA. After being washed to remove adherent bacteria, the cells were fixed, permeabilized, and stained with anti-E. coli (red) antibodies, anti-vMLL5 (green) antibodies, and nuclear staining (blue). Bars, 10 μm. (E) CFU-based quantification of adherent E. coli transformed with the vector (white bars) or pScaA (gray bars) in HeLa cells that were not transfected or transfected with DNA encoding vMLL5. The data are from three independent assays. Error bars show standard deviations (SD). ***, *P < *0.001.

To confirm the physical interaction between ScaA and vMLL5, we performed an *in vitro* GST pulldown assay. We cloned and purified the extracellular domain of TSA56, spanning amino acids 19 to 455 (TSA56_19–455_), a major outer membrane protein of O. tsutsugamushi (1) and the passenger domain of ScaA, ScaA_30–1000_ (21), as GST fusion proteins using an E. coli expression system. The purified recombinant proteins were incubated with HEK293T cell lysate expressing full-length vMLL5 and analyzed by Western blotting using anti-HA after the pulldown assay. We observed that vMLL5 bound specifically to GST-ScaA but not to GST or GST-TSA56 (Fig. 2A, left). The ScaA derived from three different prototypes (Karp, Kato, and Gilliam) of O. tsutsugamushi also bound to vMLL5 (Fig. 2A, right). This result suggests that vMLL5 is a common receptor for ScaA ligands from various O. tsutsugamushi genotypes.

To examine whether O. tsutsugamushi colocalizes with vMLL5 during the infection process, we transfected HeLa cells with a plasmid expressing vMLL5 and infected them with O. tsutsugamushi for 15 min. Bacteria were stained with anti-ScaA antibody, and cellular vMLL5 was stained with anti-Flag antibody and then analyzed using a confocal microscope. When we analyzed 100 ScaA-stained bacterial particles at the cell surface (white arrow) data from multiple images (Fig. 2B; Fig. S2), all the O. tsutsugamushi organisms at the cell surface were colocalized with overexpressed vMLL5. To further confirm the specific interaction of O. tsutsugamushi with endogenous vMLL5 protein during the infection process, we incubated human umbilical vein endothelial cells (HUVECs) with O. tsutsugamushi for 15 min and stained them with anti-ScaA and anti-MLL5 antibodies. Fluorescence-labeled phalloidin was used to visualize actin cytoskeletons that are reorganized in the membrane protrusions at the sites of infection during intracellular invasion by O. tsutsugamushi (15). We observed consistent colocalization of ScaA and endogenous MLL5 on the cellular surface (Fig. 2C; Fig. S3).

10.1128/mbio.01543-22.2 FIG S2 Immunofluorescence confocal microscopy of infected HeLa cells overexpressing vMLL5 showed that O. tsutsugamushi (red for ScaA) colocalizes with vMLL5 (green, Flag tagged) at the cell surface. Bars, 5 μm. Download FIG S2, PDF file, 0.5 MB. Copyright © 2022 Nguyen et al. 2022 Nguyen et al. https://creativecommons.org/licenses/by/4.0/ This content is distributed under the terms of the Creative Commons Attribution 4.0 International license.

10.1128/mbio.01543-22.3 FIG S3 (A) Immunofluorescence confocal microcopy of infected HUVECs showed that O. tsutsugamushi (red) colocalized with endogenous MLL5 (cyan). Actin mobilization surrounding invading O. tsutsugamushi (orange arrow) was visualized by phalloidin staining (green). White arrow, adherent O. tsutsugamushi without inducing actin reorganization. Bars, 10 μm. (B) MCCs of ScaA with vMLL5 signal. M1 indicates the fraction of vMLL5 overlapping ScaA, and M2 indicates the fraction of ScaA overlapping vMLL5 (M1, 0.53 ± 0.12; M2, 0.49 ± 0.13 [40 images]). (C) Comparison of normalized fluorescence intensities of actin signal from circular ROI of O. tsutsugamushi particles with (a; *n *= 62) or without (b; *n *= 156) actin mobilization. Mean fluorescence intensity of actin in invading O. tsutsugamushi (a) was 4.2 times higher than that of bacterial particles without actin recruitment (b). ****, *P* < 0.0001 (unpaired Mann-Whitney test). Download FIG S3, PDF file, 0.5 MB. Copyright © 2022 Nguyen et al. 2022 Nguyen et al. https://creativecommons.org/licenses/by/4.0/ This content is distributed under the terms of the Creative Commons Attribution 4.0 International license.

Quantification of 218 ScaA-positive bacterial particles associated with host cells in 40 microscopic images data revealed that 100% of O. tsutsugamushi were costained with MLL5 (Fig. 2C, white and orange arrows; Fig. S3A). Manders’ colocalization coefficients (MCCs) for ScaA with vMLL5 signals were approximately 0.5, due to partial overlapping of vMLL5 with ScaA-positive O. tsutsugamushi particles (Fig. S3B). Interestingly, the colocalization of both proteins was detected not only in invading O. tsutsugamushi, which accompanied actin reorganization on the cellular surface, but also in adherent bacteria that bound to the cellular surface without inducing actin mobilization in the nonphagocytic host cells. Approximately 28% of them were also associated with actin reorganization in host cells, as revealed by enhanced actin signal surrounding the bacterial particles (Fig. S3C, orange arrow). This suggests that ScaA may simply mediate the interaction with host MLL5 in the adhesion step, but this may not be sufficient to induce the host cellular signaling required for actin reorganization and membrane ruffling at the site of O. tsutsugamushi invasion (15).

To further investigate whether vMLL5 enhances bacterial adherence, we utilized a heterologous E. coli expression system that encodes full-length ScaA (pScaA) (21). HeLa cells overexpressing vMLL5 were incubated with recombinant E. coli harboring an empty vector or pScaA. Then, the cells were washed extensively to remove nonadherent bacteria and subsequently fixed and analyzed under a confocal microscope. As a result, vMLL5 and pScaA E. coli were colocalized at the cell surface (Fig. 2D). E. coli expressing ScaA showed significantly enhanced bacterial adherence to normal HeLa cells compared with control E. coli (Fig. 2E), as we reported previously (21). Overexpression of vMLL5 in host cells further enhanced bacterial adhesion of pScaA E. coli, but enhanced binding was not observed in control bacteria that did not express ScaA (Fig. 2E). This clearly demonstrates that the specific interaction of ScaA with host vMLL5 facilitates bacterial adhesion to host cells.

### Mapping of the vMLL5-ScaA interaction domain.

**FIG 3:**
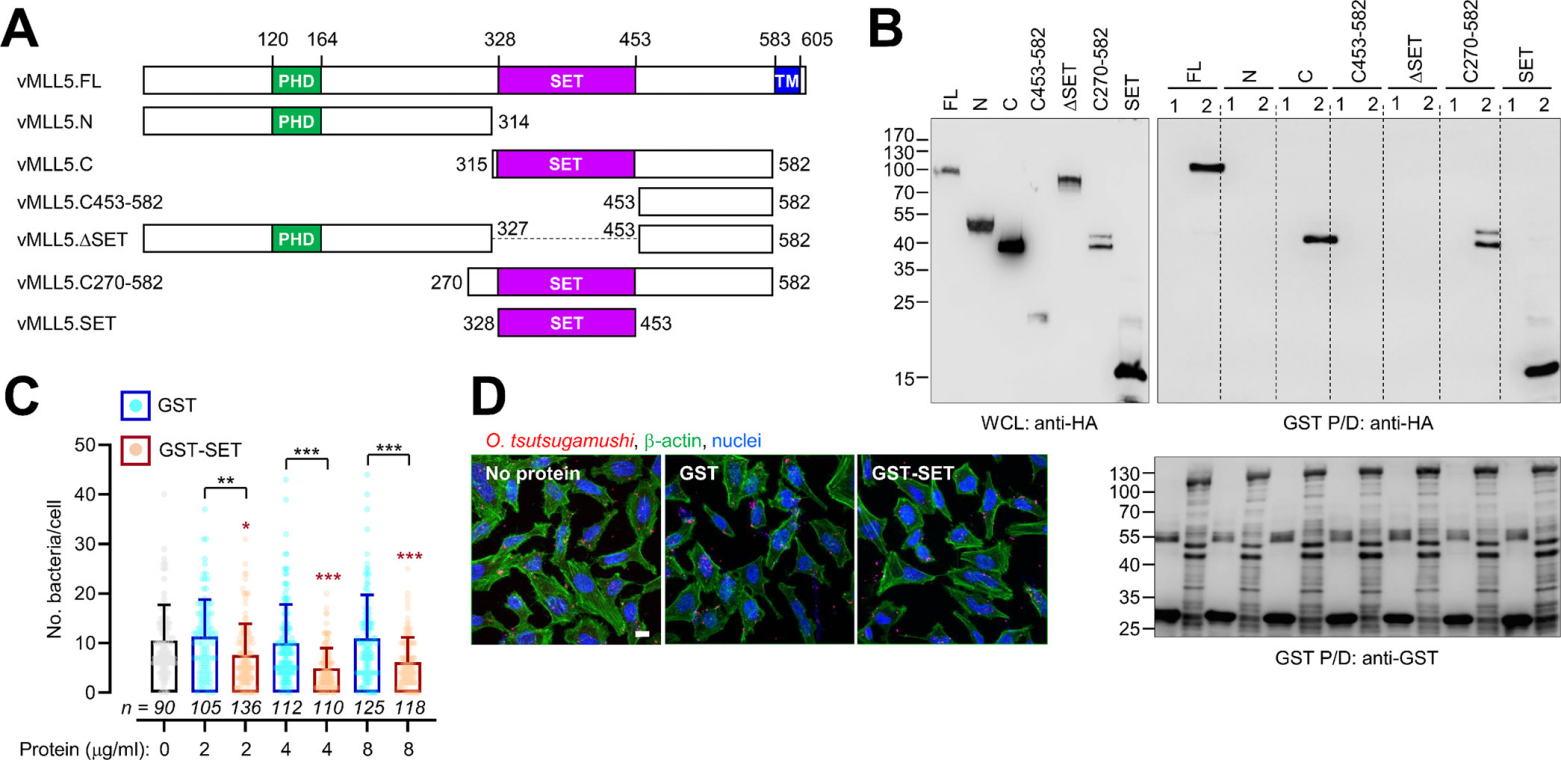
Identification of the ScaA-interacting motif in vMLL5. (A) Schematic representation of the truncated vMLL5 mutants used in this study. (B) The physical interaction of the truncated vMLL5 mutants (HA tagged) with ScaA was examined by *in vitro* GST pulldown assays using GST or GST-ScaA. HeLa cell lysates expressing the indicated vMLL5 mutants were incubated with purified GST or GST-ScaA, and the interacting vMLL5 mutants were detected by immunoblotting with anti-HA antibody. FL, full-length protein; N, N terminus; C, C terminus; WCL, whole-cell lysate; P/D, pulldown. (C) Effect of a vMLL5 SET domain on cellular adhesion of O. tsutsugamushi in HeLa cells. O. tsutsugamushi was preincubated with increasing concentrations (2 to 8 μg/mL) of purified GST or GST-SET domain fusion protein at 4°C for 30 min, followed by incubation with HeLa cells at 37°C for 15 min. Then, the cells were extensively washed, and the number of host cell-associated O. tsutsugamushi bacteria per cell was determined by confocal microscopy. Host cell-associated bacteria were enumerated, as indicated by the numbers of cells, in several different fields of confocal images, and data are the average (and SD) number of bacteria per host cell. **, *P < *0.01; ***, *P < *0.001. (D) Representative confocal images obtained from a competitive inhibition assay of O. tsutsugamushi interaction with host cells using GST or GST-SET polypeptides. Bars, 10 μm.

To define the domain of binding of vMLL5 to ScaA, we performed a GST pulldown assay using various truncated mutants of HA-tagged vMLL5 (Fig. 3A). Each vMLL5 mutant construct was transfected into HEK293T cells, and its expression was confirmed by Western blotting (Fig. 3B). An *in vitro* GST pulldown assay using GST or GST-ScaA revealed that the C-terminal region of vMLL5 (vMLL5.C), including the SET domain, specifically bound to ScaA, whereas the N-terminal counterpart (vMLL5.N) failed to interact with ScaA. To further define the ScaA binding region in vMLL5, an additional pulldown assay using various truncated constructs of vMLL5 was performed, and the results showed that the SET domain was essential and sufficient for specific interaction with ScaA (Fig. 3B).

We further investigated whether the soluble SET domain of vMLL5 could competitively inhibit the interaction of O. tsutsugamushi with host cells. O. tsutsugamushi was preincubated with the indicated amounts of soluble GST or GST-SET domain polypeptide at 4°C for 30 min, followed by incubation with HeLa cells at 37°C for 15 min. Then, the cells were extensively washed, and the number of host cell-associated O. tsutsugamushi organisms per cell was determined by confocal microscopy. As shown in Fig. 3C and D, preincubation of the bacteria with GST-SET protein induced a significant decrease in the number of bacteria per cell compared with those of controls incubated with GST. The number of bacteria per cell was reduced by approximately 33 to 51% after incubation with the GST-SET polypeptide compared with those of the GST-treated group, depending on the concentration (2 to 8 μg/mL) of the soluble GST-SET domain incubated. This suggests that the saturating concentration (~4 μg/mL) of GST-SET polypeptide could competitively inhibit the pathogen binding to host cells by approximately 50%.

**FIG 4:**
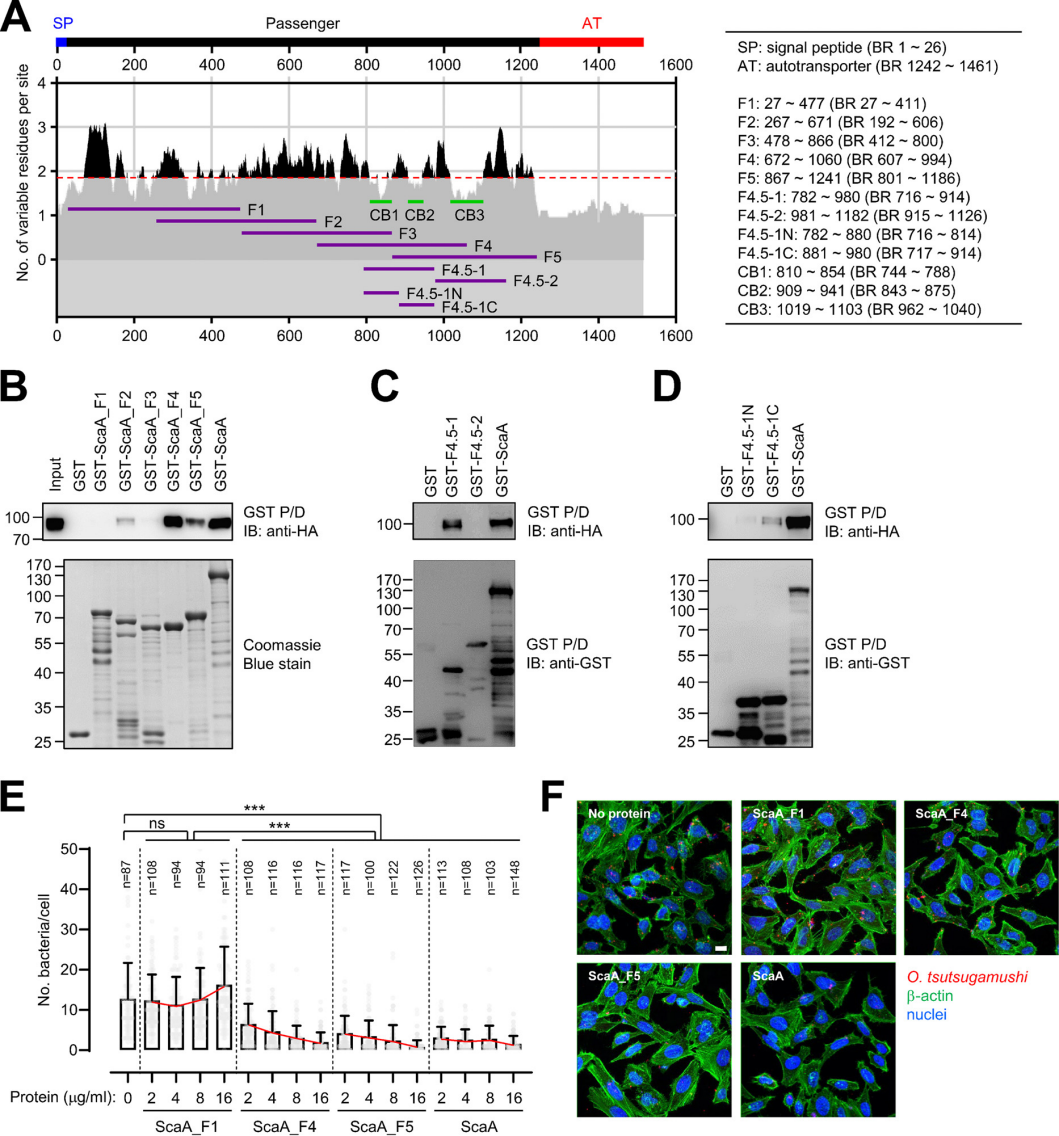
Identification of the vMLL5-binding motif in ScaA. (A) Schematic representation of the ScaA fragments used in this study. The plot shows the moving mean for a window of 10 amino acid residues of the number of different amino acids observed at a position in the amino acid alignment using 15 *scaA* genes (Table S2). The overall average of sequence variation is noted by the red dashed line. The amino acid position (aligned position and in the Boryong genotype) of each fragment is summarized on the right. CB, conserved block presenting a lower amino acid variation per site in F4 and F5 fragments compared with the overall average of sequence variation. (B to D) Interaction of truncated ScaA proteins and vMLL5. HeLa cell lysate expressing vMLL5 (HA tagged) was incubated with the indicated GST-tagged truncated ScaA proteins and used for a GST pulldown assay, followed by immunoblotting with anti-HA antibody. (E) Effect of ScaA fragments on cellular adhesion of O. tsutsugamushi in HeLa cells. Host cells were preincubated with increasing concentrations (2 to 16 μg/mL) of ScaA fragment proteins for 30 min, followed by infection with O. tsutsugamushi at 37°C for 15 min. Then, the cells were extensively washed, and the number of host cell-associated O. tsutsugamushi organisms per cell was determined by confocal microscopy. Host cell-associated bacteria were enumerated, as indicated by the numbers of cells, in several different fields of confocal images, and data are the average (and SD) number of bacteria per host cell. ns, not significant; ***, *P < *0.001. (D) Representative confocal images obtained from competitive inhibition assay of O. tsutsugamushi interaction with host cells using the indicated polypeptides. Bars, 10 μm.

Next, we tried to define the specific region of ScaA that interacts with vMLL5. GST pulldown assays were performed using truncated fragments of ScaA proteins fused with GST (Fig. 4A) and HEK293T cell lysate expressing full-length vMLL5. GST-ScaA_F4 and F5, encompassing the C-terminal region of the ScaA passenger domain, interacted strongly with vMLL5, whereas GST-ScaA fragments containing the N-terminal region of ScaA failed to interact with (ScaA_F1 and F3) or showed weak binding to (ScaA_F2) the host protein (Fig. 4B). These results suggest that the overlapping regions of ScaA_F4 and ScaA_F5 may be involved in the specific interaction with vMLL5. We further generated truncated mutants of ScaA including the overlapping sites and identified the region required for vMLL5-binding. The ScaA_F4.5-1 region strongly interacted with vMLL5, and the ScaA_717–914_ fragment (GST-ScaA_F4.5-1C) retained binding capacity for vMLL5 (Fig. 4C and D). When we assessed the amino acid sequence variation of ScaA proteins from 15 different genotypes of O. tsutsugamushi, amino acids 717 to 914 of ScaA from the Boryong genotype included a relatively conserved region, CB2, among the various ScaA proteins (Fig. 4A and Table S2). The sequence conservation among various genotypes and binding capacity to vMLL5 further support the idea that the C-terminal region of ScaA might be a strong candidate region for interaction with vMLL5, potentially through SET domain binding.

10.1128/mbio.01543-22.6 TABLE S2 Aligned amino acid sequences of 15 ScaA proteins from various O. tsutsugamushi genotypes. Download Table S2, XLSX file, 0.1 MB. Copyright © 2022 Nguyen et al. 2022 Nguyen et al. https://creativecommons.org/licenses/by/4.0/ This content is distributed under the terms of the Creative Commons Attribution 4.0 International license.

We also investigated whether the recombinant ScaA fragments with affinity for vMLL5 would inhibit cellular association of O. tsutsugamushi. The average number of bacteria per cell was significantly reduced in the presence of ScaA_F4, ScaA_F5, and the full-length ScaA passenger domain, whereas ScaA_F1, which failed to interact with vMLL5, was unable to reduce the bacterial association with host cells (Fig. 4E and F). The presence of increasing concentrations of recombinant ScaA fragments, ScaA_F4 and F5, in infection medium showed a dose-dependent reduction in the average number of O. tsutsugamushi organisms associated with HeLa cells. In contrast, the bacterial association of host cells was barely affected in the presence of ScaA_F1 fragment and presented bacterial counts per cell similar to those detected in the infection condition without exogenous protein. The average reduction rate in the number of O. tsutsugamushi bacteria per cell ranged from 51% to 84% in the presence of ScaA_F4, 67% to 93% for ScaA_F5, and 75% to 88% for the full ScaA passenger domain when the proteins were present at concentrations of 2 to 16 μg/mL. Collectively, the C-terminal fragments of the ScaA passenger domain, ScaA_F4 and F5, including a conserved amino acid sequence (CB2), were sufficient for vMLL5 interaction and inhibition of cellular binding of O. tsutsugamushi.

### Immunization of vMLL5-binding ScaA fragments for efficient protective immunity against O. tsutsugamushi infection.

**FIG 5:**
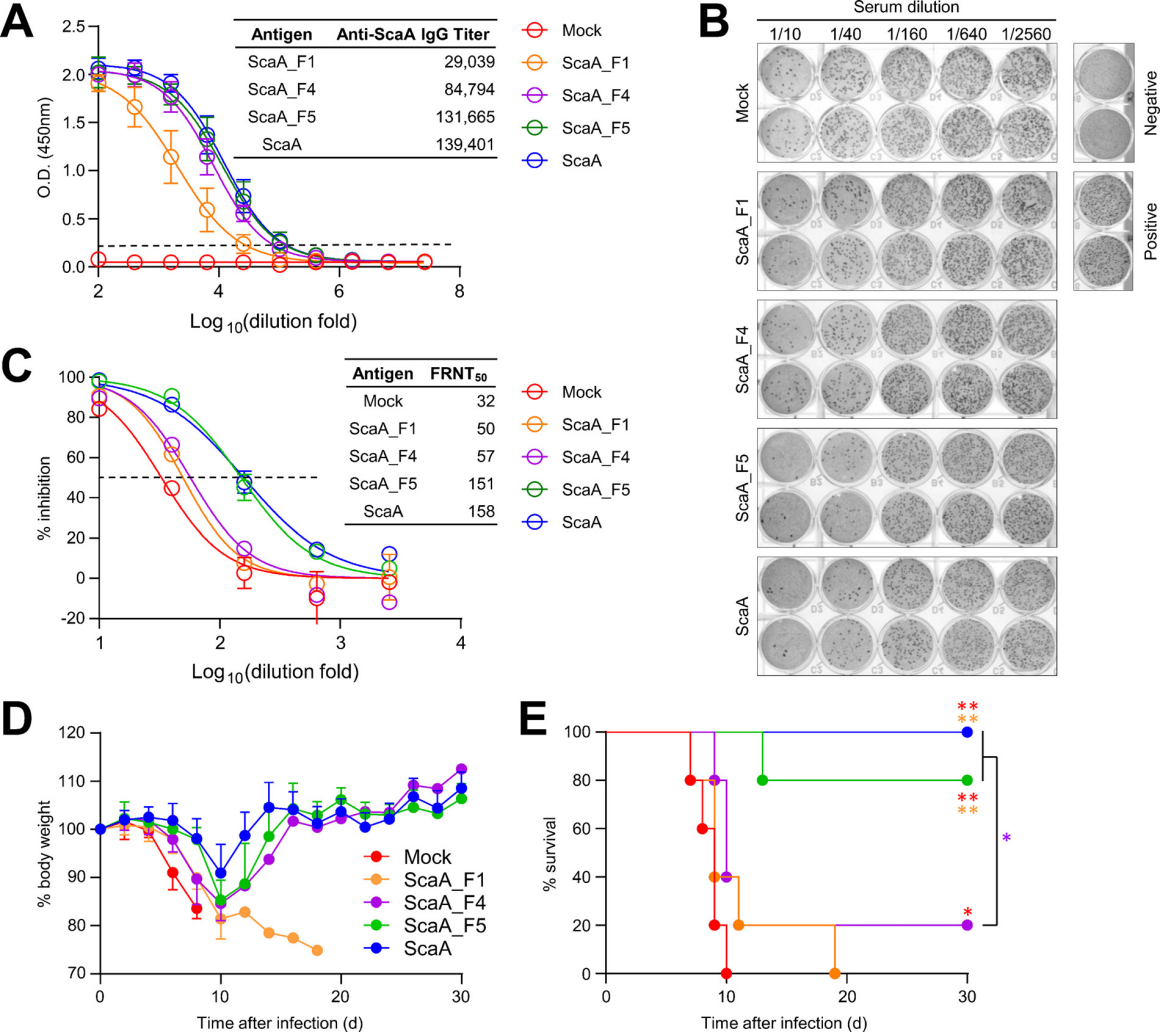
Protective role of vMLL5-binding ScaA fragment antigens against lethal O. tsutsugamushi challenge. (A) ScaA-specific IgG responses in immune sera were measured by ELISA at 1 week after the third immunization. Cutoff titers (dashed line, mean optical density [O.D.] + 3 SD at a 1:100 dilution) were determined using preimmune sera. The calculated mean antibody titers from immunized mice (5/group) are summarized in the table. (B and C) Neutralizing antibody titers (FRNT_50_) of pooled sera from immunized mice were determined by an *in vitro* FRNT assay (duplicate experiments). (B) Bacterial foci stained with anti-O. tsutsugamushi serum. Negative, uninfected wells; positive, infected wells without sera. (C) Percent inhibition. The calculated FRNT_50_ of pooled sera from the indicated group of immune mice are summarized in the table. (D and E) Mice (5/group) were immunized with the indicated antigens and challenged intravenously with 10 LD_50_s of O. tsutsugamushi. Changes in body weight (D) and the survival rate (E) of the mice were observed for 30 days after infection. *, *P < *0.05; **, *P < *0.01.

We previously showed that the anti-ScaA antibody could neutralize bacterial adhesion to host cells and that vaccination with ScaA antigen provided protective immunity against lethal infection with O. tsutsugamushi (21). Thus, we tested whether immunization with vMLL5-interacting ScaA regions provides protective immunity against O. tsutsugamushi challenges. We expressed recombinant ScaA fragments in E. coli, purified them (Fig. S4), and subcutaneously immunized the mice three times with the indicated recombinant antigen (10 μg/mouse) mixed with an adjuvant (monophosphoryl lipid A [MPLA] plus Quil-A). One week after the third immunization, we collected sera from immunized mice to examine the ScaA-specific antibody titers and their neutralizing activities. Although the antibody titers were variable depending on the immunizing antigens, we observed a significant induction of ScaA-specific IgG antibodies (Fig. 5A). The mean titer of antibody against the full-length ScaA passenger domain was highest in mice immunized with the whole ScaA passenger domain antigen (1:139,401). ScaA_F5 immunization also achieved similarly high antibody titers (1:131,665). Immunization with ScaA_F4 induced a lower specific-antibody titer (1:84,784) than that induced by ScaA_F5, and ScaA_F1 antigen induced the lowest antibody titer (1:29,039) against the ScaA passenger domain. This indicated that ScaA_F5 retains the highest immunogenicity among the truncated ScaA antigens and is capable of inducing antibody titers equivalent to those generated by the full passenger domain antigen.

10.1128/mbio.01543-22.4 FIG S4 Purified ScaA proteins used for the immunization study. Download FIG S4, PDF file, 0.2 MB. Copyright © 2022 Nguyen et al. 2022 Nguyen et al. https://creativecommons.org/licenses/by/4.0/ This content is distributed under the terms of the Creative Commons Attribution 4.0 International license.

We then measured the neutralizing activity against O. tsutsugamushi in pooled immune sera collected from the groups of mice and determined 50% focus reduction neutralization titers (FRNT_50_) (Fig. 5B and C). FRNT_50_ was the highest in the ScaA immune sera (1:158), and pooled sera from mice immunized with ScaA_F5 showed similar FRNT_50_ (1:151). These results were approximately five times higher than that (1:32) of nonimmune sera from mock-immunized mice. However, immune sera against ScaA_F4 (1:57) and ScaA_F1 (1:50) exhibited lower neutralizing activity, and the FRNT_50_ were lower by ~65% than those of ScaA and ScaA_F5 immune sera. Finally, we challenged the vaccinated mice intravenously with 10× the 50% lethal dose (LD_50_) of O. tsutsugamushi 2 weeks after the third immunization. All the mock-immunized mice rapidly lost weight and died within 10 days after infection, whereas the mice immunized with ScaA or ScaA_F5 showed delayed weight loss and significantly enhanced survival rates (100% or 80%, respectively). All the mice vaccinated with ScaA_F1 died after the lethal challenge with O. tsutsugamushi, and only one mouse immunized with ScaA_F4 survived (survival rate, 20%). Notably, the morbidity and mortality of immunized mice after lethal challenge were correlated with the neutralizing activity of immune sera against O. tsutsugamushi infection. Taken together, these results showed that immunization with the ScaA fragment ScaA_F5, which has binding affinity for the vMLL5 host receptor, is capable of generating enhanced neutralizing activity and provides significant protection against lethal O. tsutsugamushi infection comparable to that of vaccination with the full-length ScaA passenger domain.

## DISCUSSION

O. tsutsugamushi is an obligately intracellular bacterium that must be internalized in host cells to manipulate cellular machinery to promote its own replication. Even though the bacterium can infect and proliferate in phagocytic leukocytes, such as dendritic cells, macrophages, and polymorphonuclear leukocytes (26–28), infection of nonphagocytic cells, such as endothelial cells and cardiac myocytes, has been also acknowledged to support bacterial proliferation and/or disease progression in patients with severe scrub typhus (26, 29, 30). Nevertheless, the precise mechanism of host cell invasion, especially in nonphagocytic host cells, has been poorly defined. Our group has reported the host receptor and bacterial ligand interactions required for host cell binding and the cellular invasion process (14, 15, 19–21). We previously demonstrated that TSA56, a major outer membrane protein of O. tsutsugamushi, binds to fibronectin and facilitates bacterial entry into the host cell, potentially via an interaction with host integrins (14). Invading O. tsutsugamushi colocalizes with integrin α5β1 and activates integrin signaling effectors, including focal adhesion kinase, Src kinase, and RhoA GTPase, as well as signaling adaptors, such as talin and paxillin, to the site of infection (15). These invading processes also induce substantial actin reorganization and membrane protrusions at the sites of infection of nonphagocytic host cells (15).

We also showed that a conserved autotransporter protein, ScaC, mediates bacterial adhesion to host cells, potentially via fibronectin binding (19). In addition, another autotransporter protein of O. tsutsugamushi, ScaB, promotes bacterial invasion into host cells (20). However, the *scaB* gene is present in limited members of O. tsutsugamushi genotypes (17), and its potential role in differential virulence depending on genotype need to be further investigated. ScaA protein was identified as a conserved bacterial adhesin of O. tsutsugamushi (17, 21), and this autotransporter antigen is capable of inducing neutralizing activity against O. tsutsugamushi after immunization and provides significant protection against lethal infection (21, 31). However, the molecular structure of the extracellular passenger domain of ScaA is poorly defined, and its host cell receptor is yet to be elucidated. Given that multiple membrane proteins of O. tsutsugamushi are functionally involved in the host adhesion and cellular invasion process and that they could be applied as rational candidates of vaccine antigens, understanding the specific interactions between the bacterial ligand and host receptor is a critical step for developing effective preventive measures for scrub typhus (13).

In this study, we identified a novel splicing variant of MLL5 (vMLL5) as a host receptor for ScaA using Y2H screening. The MLL5 gene, also known as the lysine *N*-methyltransferase 2 gene, was initially identified as a candidate tumor suppressor gene consisting of 25 coding exons translated into a protein of 1,858 amino acid residues (32). It contains a single plant homeodomain, Su(var)3-9, an enhancer of zeste, and a SET domain close to the N-terminal regions. MLL5 plays a role in various biological functions, including cell cycle progression, genomic maintenance, and spermatogenesis (22).

Several alternative splicing variants of MLL5, such as HsMLL5α, HsMLL5β, and NKp44L, and their functions have been reported previously (22). The HsMLL5α and HsMLL5β variants are short N-terminal isoforms of MLL5 composed of 609 and 503 amino acids, respectively. These variants are known to be strictly localized in the nucleus and induce H3K4 trimethylation of histones, resulting in activation of E2F transcription factor 1 (E2F1) target genes (25). They are known to be specifically expressed in human papillomavirus 16- and 18-positive cervical cancer cells, where they are essential for transcriptional activation of the E6 and E7 oncogenes, respectively (33). Unlike HsMLL5α and HsMLL5β, NKp44L is absent in the nucleus but is expressed at the cellular surface and cytoplasm. Its gene shares the first 20 coding exons of the MLL5 gene with an additional unique exon, exon 21, encoding a specific C-terminal motif, and it functions as a cellular ligand for NKp44 (23).

These reports prompted us to evaluate whether MLL5 is a potential host receptor for ScaA because of the different functions of MLL5 variants depending on their location. We found that the gene for the ScaA-binding vMLL5 consists of exon 1 through part of exon 13 and the following intron sequences (Fig. 1A). Interestingly, unlike the previous MLL5 variants, the newly identified vMLL5 gene is predicted to encode the transmembrane at the C terminus of the intron region and is expressed in both the plasma membrane and subcellular regions but not in the nucleus (Fig. 1C). We confirmed the colocalization of O. tsutsugamushi, as well as ScaA-expressing E. coli, and vMLL5 at the cell surface. In addition, overexpression of vMLL5 significantly enhanced the adherence of ScaA-expressing E. coli to host cells. Martinez et al. previously reported that the outer membrane protein B (OmpB) autotransporter protein from Rickettsia conorii interacts with Ku70, which functions as a host receptor for R. conorii (34). Ku70 is primarily expressed in the nucleus and is involved in numerous biological processes, including transcription regulation, chromosome maintenance, V(D)J recombination, and a specific DNA repair mechanism. However, it is also expressed in the cytoplasm and plasma membrane, where it inhibits Bax-mediated apoptosis and adherence to fibronectin, respectively (35). Thus, the exploitation of a cellular protein primarily located in intracellular compartments as a host receptor is not unusual for rickettsial pathogens.

Here, we further determined that ScaA can specifically bind to vMLL5 via the SET domain (Fig. 3). To date, numerous SET domain-containing proteins known to catalyze the methylation of histones and nonhistones have been classified according to site-specific methylation or surrounding sequences (36). However, the MLL5 SET domain lacks specific motifs that are important for methyltransferase activity, and its molecular functions remain unclear (37). Our data suggest that surface-expressing MLL5 SET domains may have functions different from those of other SET domain-containing proteins. Nevertheless, the SET-domain proteins have been found in many eukaryotic organisms, from humans to *Drosophila* and yeast (36), suggesting that this domain may be exploited by O. tsutsugamushi as an evolutionarily conserved host receptor, even in the vector mite species. A BLAST search of the SET domain sequence of vMLL5 found a histone-lysine *N*-methyltransferase NSD2-like protein of Leptotrombidium delicense (GenBank ID RWS26986.1; identity = 30%, similarity = 52%). It remains to be examined whether it provides a direct binding site for ScaA or forms a complex with other accessory proteins that induce bacterial adherence.

We previously demonstrated that anti-ScaA antibody possessed neutralizing activity against O. tsutsugamushi infection (21). Here, we further defined the target region of anti-ScaA neutralizing antibody that potentially overlapped the vMLL5-binding region located in the C terminus of the ScaA passenger domain (Fig. 4 and 5). We observed that ScaA_F4 and F5 polypeptides, which interacted with vMLL5, inhibited bacterial association with host cells as efficiently as the whole passenger domain, whereas ScaA_F1, which lacks affinity for vMLL5, failed to affect bacterial adherence to the cells. This suggests specific competition with the bacterial ligand for the pathogen interaction with host cells through the overlapping region of ScaA_F4 and F5. Indeed, we were able to isolate the interaction motif to amino acid 199 (ScaA_F4.5-1, amino acid positions 716 to 914 in the Boryong genotype). Interestingly, this binding region includes two conserved stretches of amino acids, CB1 and CB2, among various ScaA proteins from 15 O. tsutsugamushi genotypes (Fig. 4A and Table S2). A CB2-containing polypeptide, F4.5-1C (amino acid positions 843 to 875 in the Boryong genotype), displayed enhanced affinity to vMLL5 compared with F4.5-1C including CB1, suggesting that the conserved motif of CB2 might be the minimum unit for vMLL5 interaction.

**FIG 6:**
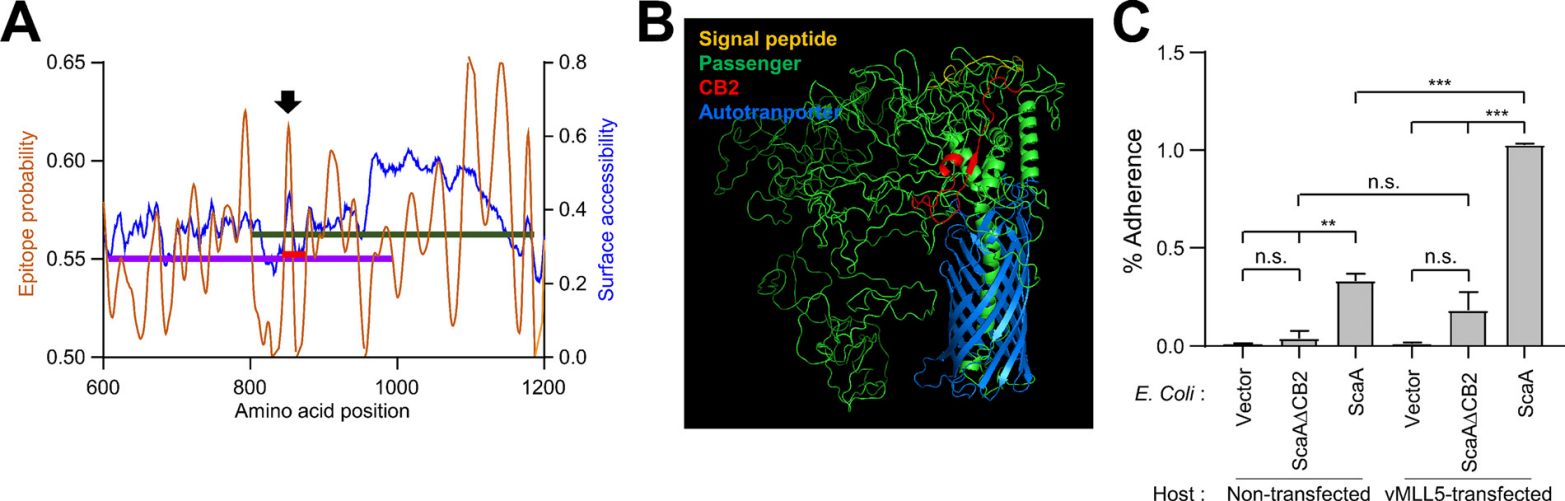
Computational prediction of B-cell epitopes, surface probability, and 3D structure of ScaA. (A) Amino acid sequences covering ScaA_F4 (purple line), ScaA_F5 (green line), and CB2 (red line) fragments were analyzed to predict epitope probability (brown line) and surface accessibility (blue line) using the BepiPred 2.0 program, and the resulting data are presented as smoothing plots (see Table S3 for raw data set). Each fragment line is positioned according to the corresponding amino acids (*x* axis) and mean epitope probability (left *y* axis). The black arrow indicates peak antigenicity and surface probability located in the N-terminal region of CB2. (B) The 3D structure of ScaA protein was predicted using the I-TASSER program. The indicated domains and CB2 motif are colored accordingly. (C) CFU-based quantification of adherent E. coli transformed with the vector pScaAΔCB2 (encoding ScaA with CB2 deleted) or pScaA (encoding wild-type ScaA) was performed in HeLa cells that were not transfected or transfected with DNA encoding vMLL5. The data are from duplicate assays. Error bars show SD. **, *P < *0.01; ***, *P < *0.001; n.s., not significant.

Computational prediction of surface accessibility and 3D modeling of ScaA protein showed that amino acids 845 to 861 (NKGSSTNPKKFDKKPV in the Boryong genotype) seem to be exposed on the protein surface (Fig. 6). In addition, this sequence motif is predicted to have a higher B cell epitope probability than the surrounding amino acids (Fig. 6A and Table S3). Nevertheless, we found that immune sera generated by immunization with ScaA_F5 showed a higher titer of antibody against the whole ScaA passenger domain and greater neutralizing activity against the bacterial pathogen than those obtained by ScaA_F4 vaccination (Fig. 5A to C), even though both fragments include the CB2 motif. Computational prediction also suggested that ScaA_F5, especially in the C-terminal region adjacent to the autotransporter domain, has more immunogenic B-cell epitopes than ScaA_F4 (Fig. 6A). In addition, the ScaA passenger domain has few predicted secondary structures, and the majority of the extracellular domain may not form a stable structure, but CB2 and the C-terminal region near the autotransporter domain possess a higher propensity to form helical domains (Fig. 6B and Table S3). Indeed, deletion of CB2 motif in ScaA failed to significantly enhance adherence of recombinant E. coli expressing the mutant ScaA (ScaAΔCB2, with deletion of 43 amino acids of CB2) to normal HeLa cells and vMLL5-overexpressing HeLa cells (Fig. 6C). We may need further analysis of the immunogenicity and neutralizing epitopes in the C-terminal region of the ScaA passenger domain to define a structural basis for the interaction with vMLL5.

10.1128/mbio.01543-22.7 TABLE S3 Computational prediction of B-cell epitopes, surface probability, and secondary structures of ScaA. Download Table S3, XLSX file, 0.1 MB. Copyright © 2022 Nguyen et al. 2022 Nguyen et al. https://creativecommons.org/licenses/by/4.0/ This content is distributed under the terms of the Creative Commons Attribution 4.0 International license.

Finally, we demonstrated that ScaA_F5 provided protective immunity against lethal challenge with O. tsutsugamushi administered intravenously as efficient as immunization with the whole ScaA passenger domain (Fig. 5D and E). The degree of protection provided by immunization with ScaA fragments tends to be correlated with their neutralizing activity, suggesting the importance of neutralizing antibodies that block the interaction with host cells. Even though we could not exclude a potential contribution of T-cell epitopes present in the ScaA fragments (38), ScaA_F5 is the most potent antigenic proportion as a vaccine candidate, at least based on its efficient neutralizing activity. We recently demonstrated that a conserved block of TSA56 (cTSA56) confers significantly enhanced protection against heterologous-genotype infection by inducing Ag-specific T cells (38). Thus, further studies evaluating the vaccine efficacy of recombinant antigens, including both cTSA56 and the vMLL5-binding motif of ScaA, would be valuable.

## MATERIALS AND METHODS

### Cell lines and transfection.

L929 cells (ATCC NCTC929; American Type Culture Collection), HeLa cells (ATCC CCL-2), and HEK293T cells (ATCC CRL-3216) were maintained in Dulbecco’s modified Eagle medium (DMEM; Welgene, Daegu, South Korea) supplemented with 10% heat-inactivated fetal bovine serum (FBS) (Welgene), 100 U/mL penicillin, and 100 μg/mL streptomycin (Gibco BRL) at 37°C in 5% CO_2_. HUVECs (Sigma-Aldrich) were grown in endothelial cell growth medium (CC-3121; Lonza, Basel, Switzerland). vMLL5 transfection was performed with Lipofectamine 2000 transfecting reagent (Life Technologies, Carlsbad, CA, USA) according to the manufacturer’s instructions.

### Preparation of O. tsutsugamushi.

O. tsutsugamushi was semipurified as previously described (38). Briefly, when more than 90% of the cells were infected, as determined by an indirect immunofluorescence antibody technique, the cells were collected, homogenized using a glass Dounce homogenizer (Wheaton, Inc., Millville, NJ, USA), and centrifuged at 500 × *g* for 5 min. The supernatant was stored in liquid nitrogen until use.

### Yeast two-hybrid screening.

Yeast two-hybrid (Y2H) screening with ScaA_30–1000_ (31) as bait was performed using the human aorta cDNA activation domain (AD) library (Clontech). The Saccharomyces cerevisiae strain PBN240 (Panbionet, Inc., Pohang, South Korea) was cotransformed with two hybrid plasmids: a bait plasmid, pBCT-ScaA_30–1000_, encoding a GAL4 DNA binding domain fused to ScaA cDNA, and a pACT plasmid that carries human aorta cDNA fused to GAL4AD. During the screening process, three reporter genes (*ura3*, *ade2*, and *lacZ*), each under the control of a different GAL4 binding site, were used to minimize any false positives. After sequential screening using selective medium, we obtained 12 genuine positive clones. To confirm the specific interaction between ScaA_30–1000_ and the AD prey proteins, the AD prey DNA was amplified by PCR using total DNA purified from the positive transformants. The amplified PCR fragment, together with a linearized prey vector, was reintroduced into the yeast strain PBN204 expressing BD-ScaA_30–1000_. The specific interactions were confirmed in all the transformants by determining the expression levels of URA3, ADE2, and LacZ (Fig. S1). pBCT-polypyrimidine tract-binding protein (PTB) and pACT2-PTB served as positive controls for the protein-protein interactions. pBCT (Panbionet, Inc.) and pACT2 (Clontech Laboratories, Inc.) were used as the negative controls.

### Cloning, expression, and purification of recombinant proteins.

The *scaA* genes were PCR amplified from O. tsutsugamushi genomic DNA using the specific primer pairs listed in Table S1. The amplified DNA was then cloned into pGEX4T-1 and pET28a vectors for protein expression. The *mll5* gene used in this study was synthesized after codon optimization and then amplified using the specific primer pairs listed in Table S1. The amplified DNA with a C-terminal HA tag was cloned into the pEFIRES-P vector (39) for transfection. E. coli BL21(DE3) (Novagen, Darmstadt, Germany) was transformed with the recombinant plasmids for protein expression and purification. Following induction with isopropyl-β-d-thiogalactopyranoside (IPTG) (0.1 mM; Duchefa, Zwijndrecht, Netherlands) at 16°C for 16 h, the proteins were purified using Ni-nitrilotriacetic acid His resin (Qiagen, Carlsbad, CA, USA) or glutathione-Sepharose 4B columns (GE Healthcare, Piscataway, NJ, USA) according to the manufacturer’s instructions. The purified proteins were dialyzed against phosphate-buffered saline (PBS) in an Aside-A-Lyzer dialysis cassette (Thermo Scientific, Rockford, IL, USA) at 4°C for overnight. For the animal study, purified proteins were treated with endotoxin removal columns (Thermo Scientific, Waltham, CA, USA), and endotoxin contamination was determined using the QCL-1000 kit (Lonza, Bloemfontein, South Africa) according to the manufacturer’s instructions. All protein contained <0.05 endotoxin unit (EU)/mL of endotoxin.

10.1128/mbio.01543-22.5 TABLE S1 PCR primers used in this study. Download Table S1, XLSX file, 0.01 MB. Copyright © 2022 Nguyen et al. 2022 Nguyen et al. https://creativecommons.org/licenses/by/4.0/ This content is distributed under the terms of the Creative Commons Attribution 4.0 International license.

### Immunofluorescence microscopy.

To determine the cellular location of vMLL5, HeLa cells were transfected with a plasmid encoding full-length vMLL5. At 24 h after transfection, cells were washed 3 times with PBS and fixed with 4% paraformaldehyde. Membrane permeabilization was performed using 0.2% Triton X-100 in PBS. Cells were stained with anti-Flag and anti-HA antibodies followed by incubation with Alexa Fluor 488-conjugated goat anti-mouse IgG and Alexa Fluor 594-conjugated goat anti-rabbit IgG antibodies (Invitrogen). To determine the colocalization of vMLL5 and ScaA-expressing E. coli, vMLL5-overexpressing HeLa cells were incubated with E. coli strains harboring a vector only or pET28a carrying the *scaA* gene at 37°C for 30 min (31). The incubated cells were washed extensively with PBS and fixed with 4% paraformaldehyde (Intron, Seongnam, South Korea). After permeabilization with 0.2% Triton X-100 solution, the cells were stained with anti-Flag and anti-E. coli antibodies for 1 h, followed by incubation with Alexa Fluor 488-conjugated goat anti-mouse IgG and Alexa Fluor 594-conjugated goat anti-rabbit IgG antibodies (Invitrogen).

To examine the colocalization of O. tsutsugamushi with vMLL5, HeLa cells overexpressing vMLL5 or HUVECs (Sigma-Aldrich) were infected with O. tsutsugamushi, washed with PBS, and fixed with 4% paraformaldehyde solution. After permeabilization with 0.2% Triton X-100 solution, the cells were stained with anti-ScaA mouse immune serum and anti-Flag or anti-vMLL5 antibodies (Bioworld Technology, Bloomington, IN, USA), followed by incubation with Alexa Fluor 594-conjugated goat anti-mouse IgG and Alexa Fluor 633-conjugated goat anti-rabbit IgG antibodies (Invitrogen). Actin and cellular nuclei were stained using Alexa Fluor 488-conjugated phalloidin and 4′,6-diamidino-2-phenylindole (DAPI; Thermo Fisher), respectively, in some experiments. The stained cells were examined under an Olympus FV3000 laser scanning confocal microscope (Olympus, Tokyo, Japan). Images of cell sections were analyzed and processed using the Olympus Fluoview software (Olympus).

Colocalization of O. tsutsugamushi particles with vMLL5 or actin cytoskeleton was quantified by counting the number of bacteria overlapped with vMLL5 signal or surrounded by mobilized actin in multiple microscopic images. Regions of interest (ROI) were selected, and MCCs between the ScaA and vMLL5 channels were calculated using ImageJ with the JACoP plug-in function (https://imagej.net/plugins/jacop). The background was automatically identified using Costes’ threshold regression before MCC calculation (40). Fluorescence intensities of β-actin surrounding O. tsutsugamushi were also measured using ImageJ. Circular ROIs including O. tsutsugamushi particles with a radius of 2 μm were selected and background subtracted. Then, the normalized fluorescence intensities of actin signals surrounding O. tsutsugamushi were used to confirm the fraction of bacterial particles recruiting actin filaments of host cells.

### Cellular adhesion assay.

Bacterial adhesion assays were performed as previously described (21). Briefly, E. coli harboring a vector or pET28a encoding the *scaA* gene was induced with IPTG and added to confluent monolayers of vMLL5-overexpressing cells. Portions of the bacterium-containing medium were plated to determine the number of CFU added to each host cell monolayer. Contact between bacteria and the mammalian cells was synchronized by centrifugation at 200 × *g*, and the preparations were incubated at 37°C for 30 min. After incubation, the cells were washed extensively with PBS. Then, the bacteria were liberated by incubation with 0.1% Triton X-100 in sterile water. The lysate was then plated on LB agar to enumerate the cell-associated bacteria. The results are expressed as the percentages of bacteria recovered relative to the number of bacteria in the initial inoculum.

### GST pulldown assay.

A GST pulldown assay was performed using HEK293T cell lysate that was transfected with a full-length or truncated mutant vMLL5 construct. In brief, 1 × 10^8^ cells were harvested and lysed in NP-40 buffer (50 mM Tris-HCl [pH 7.4], 150 mM NaCl, 1% NP-40, 5 mM EDTA) supplemented with a protease inhibitor cocktail (Roche, Basel, Switzerland). The postcentrifuged supernatants were incubated with GST fusion proteins that bound to glutathione beads at 4°C for 4 h. After incubation, the beads were washed four times with binding buffer (20 mM HEPES [pH 7.4], 100 mM NaCl, 1% NP-40, protease inhibitors), and the proteins associated with the beads were analyzed by SDS-PAGE and subjected to immunoblotting using an anti-HA or anti-GST antibody.

### Cellular adhesion inhibition assay.

To test the inhibitory effect of recombinant ScaA proteins on the adhesion of O. tsutsugamushi, various amounts of recombinant ScaA polypeptides were preincubated with HeLa cell monolayer for 15 min at 37°C, and the cells were infected with O. tsutsugamushi for 30 min. Then, the cells were washed with PBS three times, fixed with 4% paraformaldehyde solution, and permeabilized with 0.2% Triton X-100 solution. The cells were stained to detect adherent O. tsutsugamushi as described above.

### Neutralizing antibody assay.

To evaluate the neutralizing activity of anti-ScaA immune sera, a FRNT assay was performed using sera from immunized mice. The O. tsutsugamushi Boryong strain (multiplicity of infection, 0.01) was preincubated with serially diluted sera from immunized mice at 4°C for 1 h. The mixture of bacteria and serum was added to a monolayer of L929 cells in a 24-well plate. After incubation for 2 h, the cells were washed three times with medium and cultured under an overlay medium (DMEM supplemented with 5% FBS and 1% methylcellulose) at 37°C for 14 days. The cells were fixed with 4% paraformaldehyde solution and 100% methanol. Bacterial foci were detected using O. tsutsugamushi-immune mouse serum and goat anti-mouse IgG secondary antibody conjugated with alkaline phosphatase (Thermo Fisher Scientific). Bacterial foci were visualized by incubation with nitroblue tetrazolium–5-bromo-4-chloro-3-indolylphosphate (NBT-BCIP) solution (Roche, Mannheim, Germany). The percent focus reduction was calculated as follows: [(number of foci without antibody) − (number of foci with antibody)]/(number of foci without antibody) × 100. Then, FRNT_50_ were calculated by a nonlinear regression analysis (log[inhibitor] versus normalized response method) embedded in GraphPad Prism software v5.01 (GraphPad Software, San Diego, CA, USA).

### Enzyme-linked immunosorbent assay (ELISA).

To determine the titers of antibodies specific to ScaA in the sera of immunized mice, immunoassay plates (96-well plates; Nunc, Rochester, NY, USA) were coated with 100 μL of purified antigens at a concentration of 1 μg/mL at 4°C overnight. The plates were then blocked for 2 h at room temperature with PBS containing 5% skim milk. Sera were serially diluted 4-fold and incubated for 2 h at room temperature. Plates were washed with PBS containing 0.05% Tween 20 (PBST), and horseradish peroxidase (HRP)-conjugated goat anti-mouse IgG (Santa Cruz Biotechnology, Santa Cruz, CA, USA) was added. The mixture was incubated for 1 h at room temperature. Wells were washed with PBST and incubated with 3,3′,5,5′-tetramethylbenzidine peroxidase substrate solution (Kirkegaard & Perry Laboratories, Gaithersburg, MD, USA) for 7 min. Finally, 1 M phosphoric acid solution was added to stop the reaction. Absorbance was measured at 450 nm using a microplate reader (Beckman Coulter, Inc., Fullerton, CA, USA).

### Immunization of mice and challenges.

Animal experiments were approved by the Seoul National University Hospital Institutional Animal Care and Use Committee (SNU-190725-4-3) and performed in strict accordance with the recommendations in the national guidelines for the care and use of laboratory animals. For immunization experiments, 6- to 8-week-old female C57BL/6 mice (Orient Bio, Inc., Seongnam, South Korea) were used. Groups (*n *= 5) of mice were immunized subcutaneously three times at 2-week intervals. Ten micrograms of purified proteins in PBS containing 10 μg of MPLA and Quil-A (InVivogen) was used for each immunization. Blood was collected from immunized mice after 1 week after the third immunization to determine serum antibody titers. Two weeks after the final immunization, mice were challenged intravenously with 10 LD_50_s (2 × 10^6^ focus-forming units) of the O. tsutsugamushi Boryong strain. Body weights and survival were monitored for 1 month after bacterial challenge.

### Computational prediction of antigenicity and structure.

The domain structure of vMLL5 (GenBank accession no. BC062583.1) was predicted using SMART (Simple Modular Architecture Research Tool) software (http://smart.embl-heidelberg.de/) (41). The amino acid sequence of ScaA (NCBI accession no. CAM79168) from the O. tsutsugamushi Boryong strain was used to predict epitope probability, surface accessibility, and secondary structures using BepiPred-2.0 (https://services.healthtech.dtu.dk/service.php?BepiPred-2.0) (42). The three-dimensional (3D) structure of the ScaA protein was predicted using the I-TASSER program (https://zhanggroup.org/I-TASSER/) (43). We applied the default settings for prediction processes.

### Statistical analysis.

Data were analyzed using Graph Pad Prism 5.01 software. Statistical analysis of the experimental data was performed using the nonparametric Kruskal-Wallis test. Data are expressed as means and standard deviations. Statistical analysis of survival rates was performed using the Mantel-Cox log-rank test. A *P* value of <0.05 was considered statistically significant.
