# Supplementary material for: An Alternative Splicing Variant of the Mixed-Lineage Leukemia 5 Protein Is a Cellular Adhesion Receptor for ScaA of Orientia tsutsugamushi
Source: mBio. 2022 Dec 21;14(1):e01543-22. doi: 10.1128/mbio.01543-22 (PMC9973269; doi:10.1128/mbio.01543-22)
Supplement: FIG S1 [file mbio.01543-22-s0004.pdf]

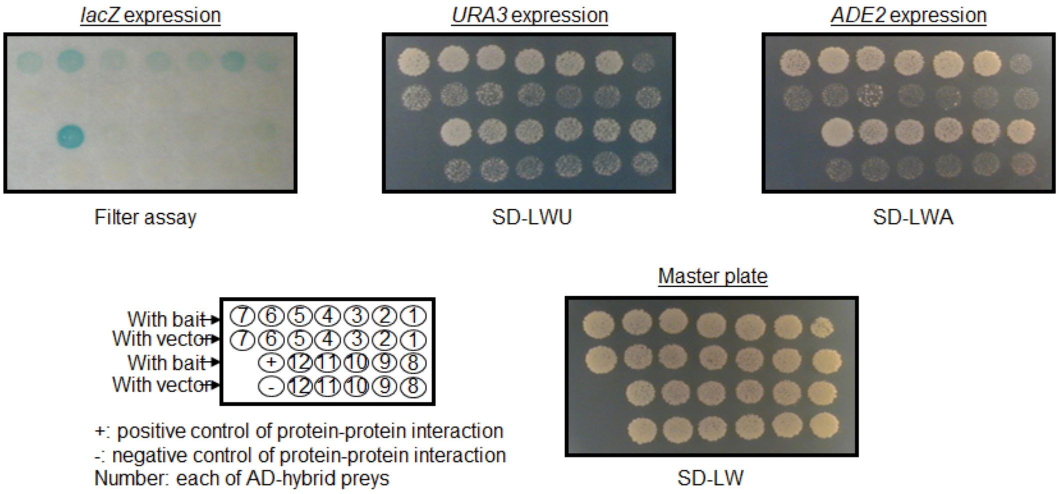

Prey ID	Description	Reporter expression		
		lacZ	*URA3*	*ADE2*
AD-Hybrid - 1	The activation domain (AD) is fused in frame to the 1941st aa of myosin, heavy chain 11, smooth muscle (MYH11), transcript variant SM2A (EU489063).	+	-	-
AD-Hybrid - 2	The activation domain (AD) is fused in frame to the 314th aa of lysine (K)-specific methyltransferase 2E (KMT2E), transcript variant 2 (NM_018682).	+	+	+
AD-Hybrid - 3	The activation domain (AD) is fused to 3' UTR (untranslated region) of nucleophosmin (nucleolar phosphoprotein B23, numatrin) (NPM1), transcript variant 2 mRNA (NM_199185).	+	+	+
AD-Hybrid - 4,5,7	The activation domain (AD) is fused to mitochondrion, complete genome (NC_012920.1).	+	+	+
AD-Hybrid - 6	The activation domain (AD) is fused to 3' UTR of serine incorporator 1 (SERINC1) mRNA (NM_020755).	+	+	+
AD hybrid - 8-11	The activation domain (AD) is fused to chromosome 19, alternate assembly CHM1_1.1 (NC_018930.2).	+/-	+	+
AD-Hybrid - 12	The activation domain (AD) is fused to chromosome 17, alternate assembly CHM1_1.1 (NC_018928.2).	-	+	+
