# Supplementary figures and images for: An Alternative Splicing Variant of the Mixed-Lineage Leukemia 5 Protein Is a Cellular Adhesion Receptor for ScaA of Orientia tsutsugamushi
Source: mBio. 2022 Dec 21;14(1):e01543-22. doi: 10.1128/mbio.01543-22 (PMC9973269; doi:10.1128/mbio.01543-22)

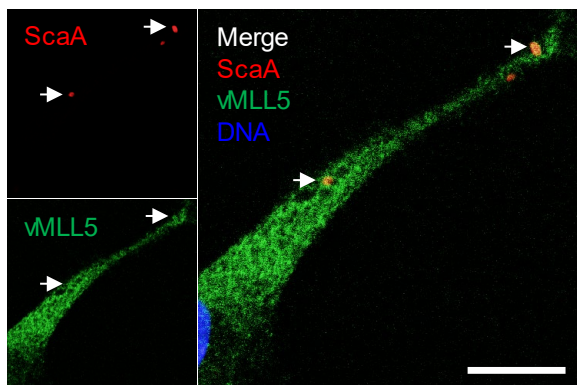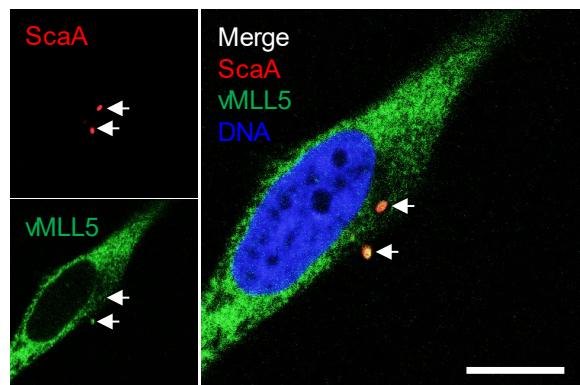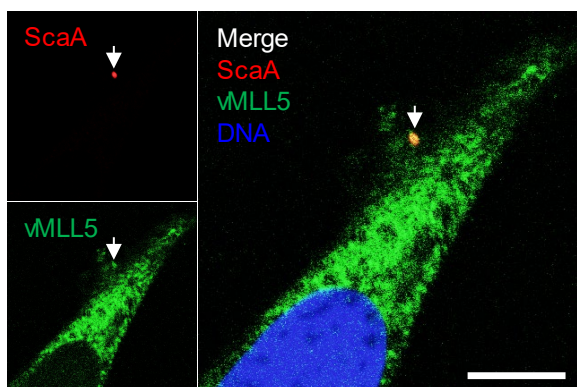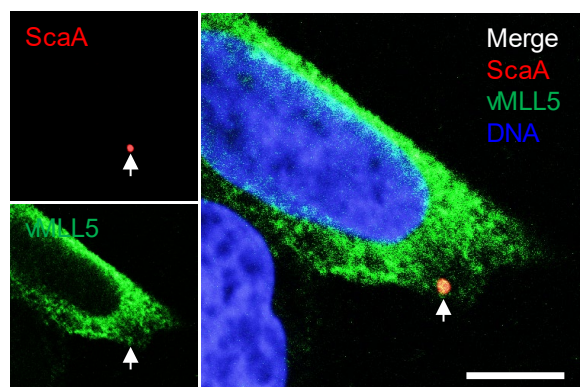

Supplement: FIG S2 [file mbio.01543-22-s0005.pdf]

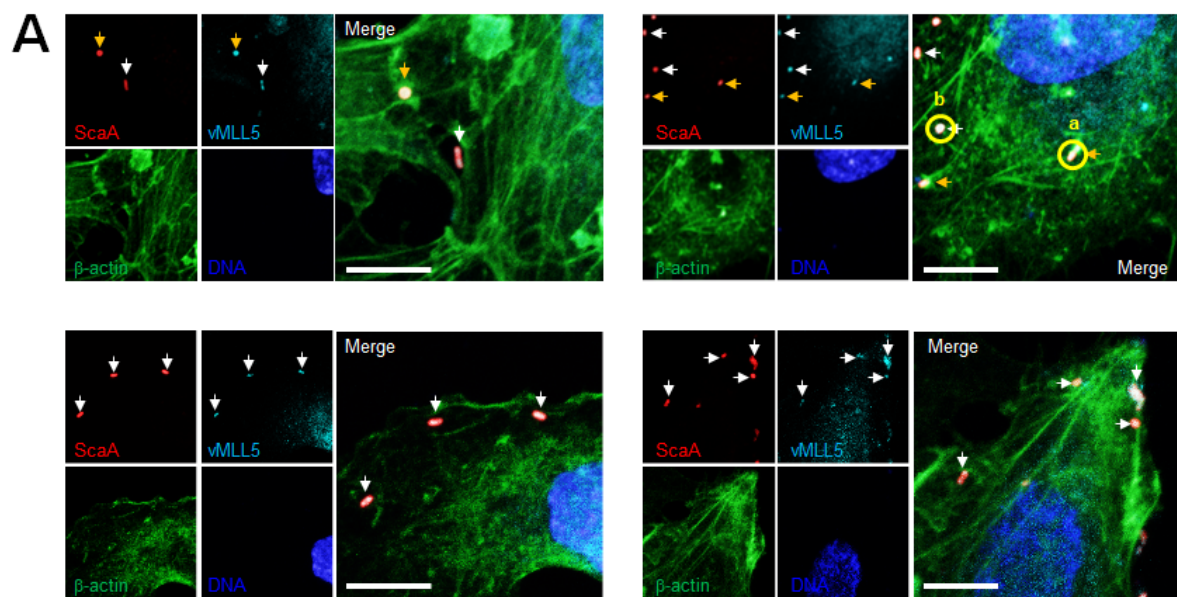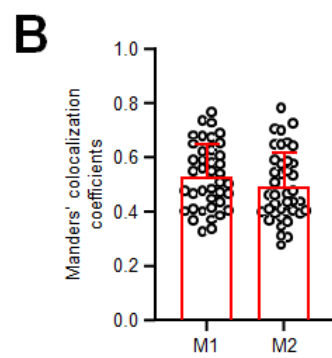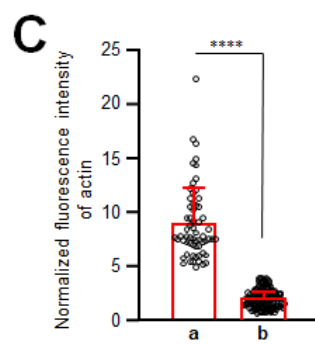

Supplement: FIG S3 [file mbio.01543-22-s0006.pdf]

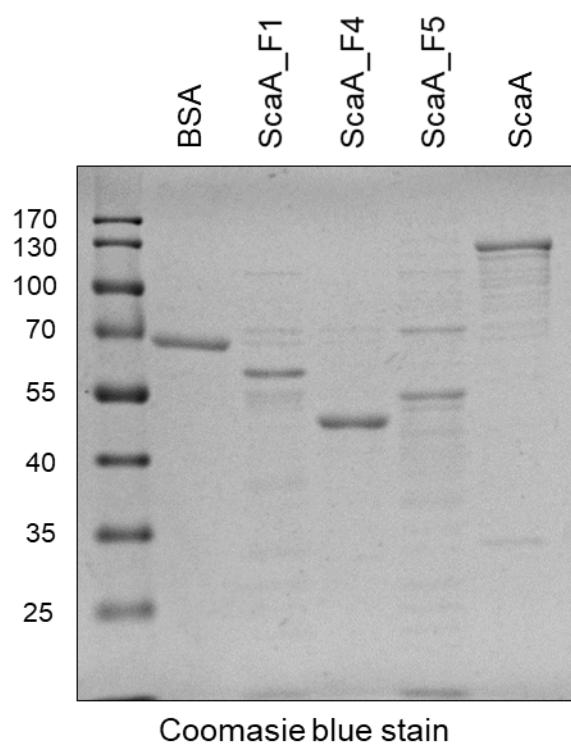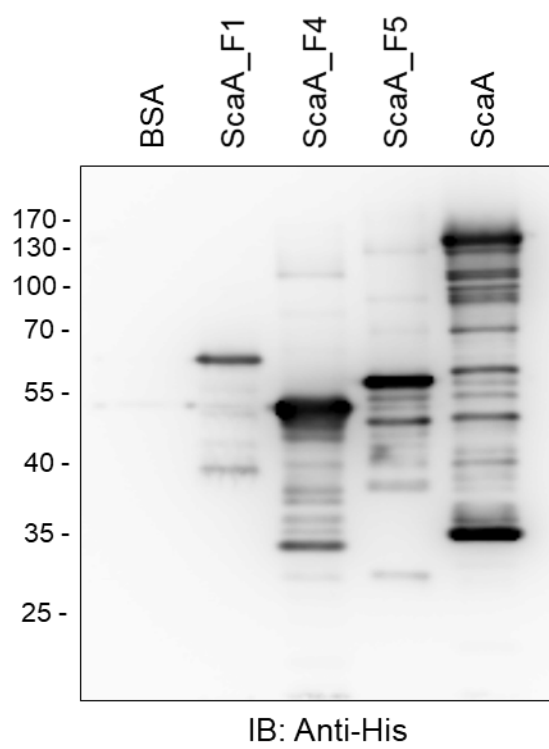

Supplement: FIG S4 [file mbio.01543-22-s0007.pdf]
